# Quinones are growth factors for the human gut microbiota

**DOI:** 10.1186/s40168-017-0380-5

**Published:** 2017-12-20

**Authors:** Kathrin Fenn, Philip Strandwitz, Eric J. Stewart, Eric Dimise, Sarah Rubin, Shreya Gurubacharya, Jon Clardy, Kim Lewis

**Affiliations:** 1 Antimicrobial Discovery Center, Department of Biology, Northeastern University, 134 Mugar Hall, 360 Huntington Ave, Boston, MA 02115 USA; 2 Department of Biological Chemistry and Molecular Pharmacology, Harvard Medical School, Boston, MA 02115 USA; 3 Present address: Department of Immunology and Infectious Diseases, Harvard T.H. Chan School of Public Health, Boston, MA 02115 USA; 4 Present address: Department of Molecular Genetics, Weizmann Institute of Science, 7610001 Rehovot, Israel

**Keywords:** Uncultured bacteria, Microbiome, Growth factors, Menaquinones, Cultivation, *Faecalibacterium*

## Abstract

**Background:**

The human gut microbiome has been linked to numerous components of health and disease. However, approximately 25% of the bacterial species in the gut remain uncultured, which limits our ability to properly understand, and exploit, the human microbiome. Previously, we found that growing environmental bacteria in situ in a diffusion chamber enables growth of uncultured species, suggesting the existence of growth factors in the natural environment not found in traditional cultivation media. One source of growth factors proved to be neighboring bacteria, and by using co-culture, we isolated previously uncultured organisms from the marine environment and identified siderophores as a major class of bacterial growth factors. Here, we employ similar co-culture techniques to grow bacteria from the human gut microbiome and identify novel growth factors.

**Results:**

By testing dependence of slow-growing colonies on faster-growing neighboring bacteria in a co-culture assay, eight taxonomically diverse pairs of bacteria were identified, in which an “induced” isolate formed a gradient of growth around a cultivatable “helper.” This set included two novel species *Faecalibacterium* sp. KLE1255—belonging to the anti-inflammatory *Faecalibacterium* genus—and *Sutterella* sp. KLE1607. While multiple helper strains were identified, *Escherichia coli* was also capable of promoting growth of all induced isolates. Screening a knockout library of *E*. *coli* showed that a menaquinone biosynthesis pathway was required for growth induction of *Faecalibacterium* sp. KLE1255 and other induced isolates. Purified menaquinones induced growth of 7/8 of the isolated strains, quinone specificity profiles for individual bacteria were identified, and genome analysis suggests an incomplete menaquinone biosynthetic capability yet the presence of anaerobic terminal reductases in the induced strains, indicating an ability to respire anaerobically.

**Conclusions:**

Our data show that menaquinones are a major class of growth factors for bacteria from the human gut microbiome. These organisms are taxonomically diverse, including members of the genus *Faecalibacterium*, *Bacteroides*, *Bilophila*, *Gordonibacter*, and *Sutterella*. This suggests that loss of quinone biosynthesis happened independently in many lineages of the human microbiota. Quinones can be used to improve existing bacterial growth media or modulate the human gut microbiota by encouraging the growth of important symbionts, such as *Faecalibacterium* species.

**Electronic supplementary material:**

The online version of this article (10.1186/s40168-017-0380-5) contains supplementary material, which is available to authorized users.

## Background

Research on the microbiome is radically changing our understanding of health and disease [1]. The gut microbiome seems to play an important role in numerous gastrointestinal and metabolic disorders, such as obesity, heart disease, type II diabetes, and cancer [2–5], and unexpectedly, the microbiome has also been linked to the nervous system and mental health disorders [6–8].

Traditionally, such microbe-disease-association studies relied heavily on cultivation, yet with the advance of metagenomics, researchers can bypass cultivation to search for links between microbial signatures and disease phenotypes [9]. This is beneficial, as many bacterial species are difficult to grow or have not yet been cultivated. However, any associations between microbiota signatures and health or disease predicted by metagenomics must be confirmed for causality using in vivo models, which requires cultivation. Cultivation efforts have improved recovery of microbiome diversity [10, 11], but according to a recent study, 23% of the 1525 bacterial species identified as residents of the human gut remain uncultured [12].

Previously, we found that growing bacteria in situ in a diffusion chamber enabled growth of uncultured bacteria [13, 14]. This suggested the existence of growth factors in the natural environment not found in traditional cultivation media. One possible source of such growth factors for uncultured bacteria is other microorganisms. By using co-culture, we found that iron-chelating siderophores produced by neighboring species were growth factors for many uncultured marine bacteria [15]. In the present study, we explored whether a similar phenomenon occurs in the human gut. Here, we report that electron-shuttling quinones, produced by cultivable bacteria, serve as required or growth-promoting factors for a variety of microorganisms of the gut microbiota.

## Results

### A screen for uncultured bacteria

**Fig. 1:**
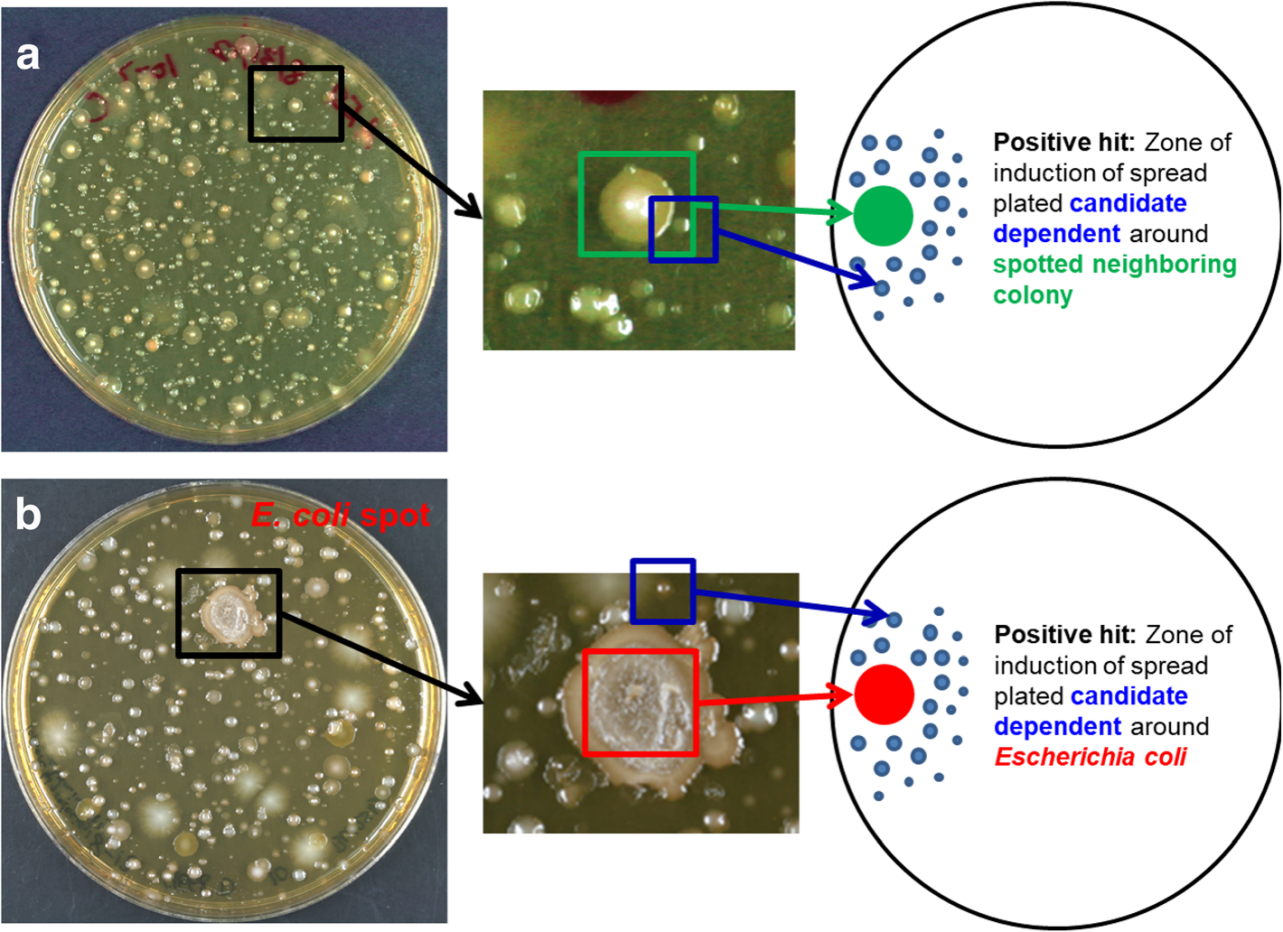
Isolating uncultured bacteria using co-culture methods. Two approaches are used to identify bacterial isolates from stool exhibiting dependency phenotypes. **a** In an untargeted approach, early-forming colonies are picked and spotted on plates where late-forming neighboring colonies are spread. **b** In a targeted approach, a known helper bacterium, in this case *Escherichia coli* K12, can be spotted at the time of inoculation of stool sample. Neighboring colonies around the *E*. *coli* spot are then picked, spread on fresh agar, and an *E*. *coli* inoculum is then spotted on the plate. In both assays, induction of the candidate-dependent bacteria around the spotted helper indicates a positive hit. The plates in this figure are used as a representative example to explain the isolation technique

We reasoned that some of the colonies that grow on nutrient agar densely plated with a human stool sample are previously uncultured bacteria, only forming because they are in close proximity to a neighboring species producing a growth factor. To explore this, we spread-plated diluted human stool to form ~ 200 colonies on a Petri plate seeded with rich agar. Bacteria that depend on growth factors from neighbors will only appear after colonies of the helper species have begun to grow. We therefore noted the slow-growing colonies (the candidate “dependent;” formation observed in 3–7 days), and screened for their dependency on faster growing neighbors (the candidate “helper;” formation observed in 1–2 days and < 5 mm away from a candidate dependent). For this, both colonies were resuspended in separate tubes, the late-forming colony was spread-plated on a fresh agar plate, and the faster growing neighbor was then spotted on the same plate (Fig. 1a). We considered a candidate dependent a positive hit if it exhibited enhanced growth around the spotted helper. Apart from this generalized approach, we also tested the ability of *Escherichia coli* to support growth of neighboring gut bacteria. An inoculum of *E*. *coli* K12 BW25113 was spotted on media spread-plated with diluted human stool, and colonies growing near the *E*. *coli* spot were then picked and screened for *E*. *coli* dependence (Fig. 1b). If growth induction was observed with *E*. *coli*, a growth factor could then be readily identified by screening a knockout library of *E*. *coli* mutants, as we showed previously for siderophores [15].

**Fig. 2:**
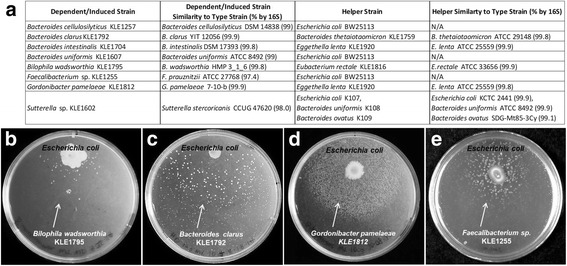
*Escherichia coli* is a universal helper for the cultured helper-dependent and helper-induced strains. All strains identified in the co-culture screen were tested for induction by *E*. *coli* K12 BW25113. Identified induced strains were spread on brain heart infusion agar with 5.0 g/L yeast extract, 0.1% cysteine, and 15 mg/mL hemin (BHIych), and 5 μL of a 24-h culture of *E*. *coli* K12 BW25113 was then spotted on the same plate. **a** Taxonomic information on the induced isolates and their helper organisms. Different growth induction profiles were identified, including complete dependence, as observed with **b** *Bilophila wadsworthia* KLE 1795, and substantially increased colony size, as seen with **c** *Bacteroides clarus* KLE1792, **d** *Gordonibacter pamelaeae* KLE1812, **e** *Faecalibacterium sp.* KLE1255, and all other isolates (not pictured). Images are displayed in black and white to improve contrast

Using these methods, several “helper-dependent” pairs were identified. After restreaking both the candidate helpers and dependents (with a nearby spot of the helper to stimulate growth) to ensure purity, 16S rRNA gene sequencing and annotation showed that the helper-dependent bacteria were taxonomically diverse, with two dependents being of low 16S rRNA gene sequence similarity to existing type strains (Fig. 2a). In total, after screening roughly 200 candidate helper-dependent pairs, we isolated eight species induced by helper strains. These are *Bacteroides clarus* KLE1792, *Bacteroides intestinalis* KLE1704, *Bacteroides cellulosilyticus* KLE1257, *Bacteroides uniformis* KLE1607, *Bilophila wadsworthia* KLE1795, *Gordonibacter pamelaeae* KLE1812, *Faecalibacterium* sp. KLE1255, and *Sutterella* sp. KLE1602 (Fig. 2a). Interestingly, while multiple helper strains were identified in the generalized screen, all isolates exhibiting dependency were also found to be induced by *E*. *coli* (examples in Fig. 2b–e). Notably, while some of the dependent organisms exhibited growth only near the spotted helper (like *B*. *wadsworthia* KLE1795, Fig. 2b), others could form smaller colonies without the presence of the helper, indicating that they can grow on their own, but at a reduced rate. Consequently, we will refer to all isolates as “induced” from here on.

Of particular interest is the previously uncultured *Faecalibacterium* sp. KLE1255, which is 97.4% similar by 16S rRNA gene sequence to *Faecalibacterium prausnitzii* ATCC 27768^T^. Members of the *Faecalibacterium* genus are generally considered to be beneficial residents of the human gut microbiome due to their anti-inflammatory properties [16], likely through induction of IL-10 [17]. *Faecalibacterium* is negatively correlated with the onset of inflammatory bowel disease [18], further suggesting an important role in human health. Therefore, we decided to isolate the growth factor of *Faecalibacterium* sp. KLE1255.

### Gene coding for the growth factor

**Fig. 3:**
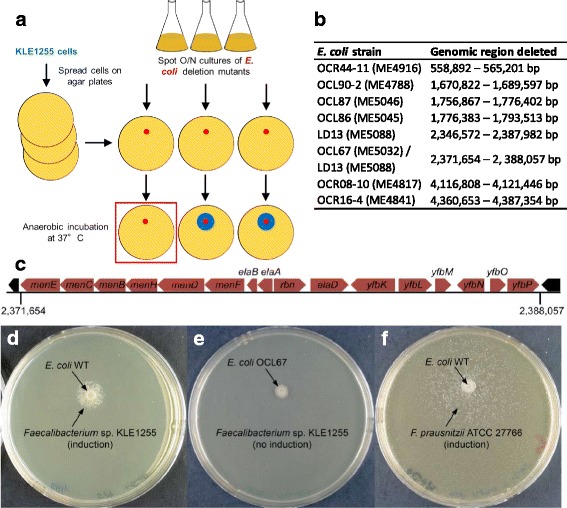
Screening for deletion mutants unable to induce the growth of *Faecalibacterium* sp. KLE1255. **a** A selection of 283 strains that were compiled from *E*. *coli* small-, medium-, and large-scale deletion libraries to cover all non-essential genes of the *E*. *coli* genome were screened for induction of KLE1255 via co-culture assay. **b** Eight strains were identified as being unable to induce growth of KLE1255, harboring deletions in various regions of the genome. **c** One strain, *E*. *coli* OCL67, had a single large deletion consisting of 16 genes, including 6 in menaquinone biosynthesis. *menE* = 2-succinylbenzoate--CoA ligase; *menC* = o-succinylbenzoate synthase; *menB* = 1,4-dihydroxy-2-naphthoyl-CoA synthase; *menH* = 2-succinyl-6-hydroxy-2,4-cyclohexadiene-1-carboxylate synthase; *menD* = 2-succinyl-5-enolpyruvyl-6-hydroxy-3-cyclohexene-1-carboxylate synthase; *menF* = isochorismate synthase; *elaB* = putative membrane-anchored DUF883 family ribosome-binding protein; *elaA* = putative N-acetyltransferase; *rbn* = ribonuclease BN; *elaD* = deubiquitinase; *yfbK* = putative lipoprotein; *yfbL* = putative membrane-associated peptidase; *yfbM* = DUF1877 family protein; *yfbN* = uncharacterized protein; *yfbO* = uncharacterized protein; *yfbP* = uncharacterized protein. **d** Wild type induced strong growth in KLE1255, while **e** OCL67 did not. Similarly, **f** WT *E*. *coli* also induces *Faecalibacterium prausnitzii* ATCC 27766

Siderophores, including enterobactin produced by *E*. *coli*, are a common growth factor for uncultured marine bacteria [15]. We also found that siderophores can be replaced by soluble Fe(II) present at high levels [15]. We therefore tested Fe(II) for its ability to induce growth of the uncultured gut bacteria we identified. However, iron had no effect on the growth of these isolates, suggesting that it is not the missing growth factor (data not shown). We then sought to identify the genes responsible for production of the growth factor and took advantage of the ordered *E*. *coli* deletion strain collections. The Keio collection is a set of 3985 knockout strains [19] that can be screened for the absence of induction, and thus lead to identification of the genetic locus responsible for production of the growth factor. To simplify the screen, we used strains with larger deletions [20, 21], and assembled a minimal set of 283 strains from small-, medium-, and large-deletion libraries to cover most non-essential genes of the *E*. *coli* genome (Additional file 1: Table S1). Strains of this library were then screened individually for induction of *Faecalibacterium* sp. KLE1255 in co-culture assays, and eight *E*. *coli* mutants were identified that did not induce growth of *Faecalibacterium* sp. KLE1255 (Fig. 3a). These mutants harbored deletions in different regions of the genome (Fig. 3b; detail on the missing genes for each strain can be found in Additional file 2: Table S2), but a general theme was an absence of genes involved with menaquinone biosynthesis. For example, one of the strains unable to induce *Faecalibacterium* sp. KLE1255, OCL67, has a single 16.4-kb region deleted from its chromosome, which consists of 16 genes, including six genes involved in menaquinone biosynthesis (Fig. 3c, e).

To narrow down the identification of relevant genes, we then tested single gene *E*. *coli* knockout mutants in the menaquinone, ubiquinone, and chorismate (the precursor to both menaquinone and ubiquinone) biosynthetic pathways from the Keio collection. Strains with deletions in the chorismate and menaquinone biosynthesis pathways were unable to induce growth of *Faecalibacterium* sp. KLE1255, suggesting that menaquinone was the growth factor for this bacterium (Additional files 3 and 4: Figures S1 and S2)*.* Its cultured relative, *F*. *prausnitzii* ATCC 27766, grows on LYHBHI, a BHI medium containing cellobiose and maltose, as well as YCFAG medium. By contrast, *Faecalibacterium* sp. KLE1255 does not grow on these media. Originally isolated in the presence of rich rumen fluid, *F*. *prausnitzii* ATCC 27766 grows poorly, which is a limitation in obtaining sufficient amounts of this symbiont. Consequently, we decided to test the ability of *E*. *coli* to stimulate growth of *F*. *prausnitzii* ATCC 27766. Interestingly, in the presence of wild type *E*. *coli*, *F*. *prausnitzii* ATCC 27766 formed noticeably larger colonies (Fig. 3f).

**Fig. 4:**
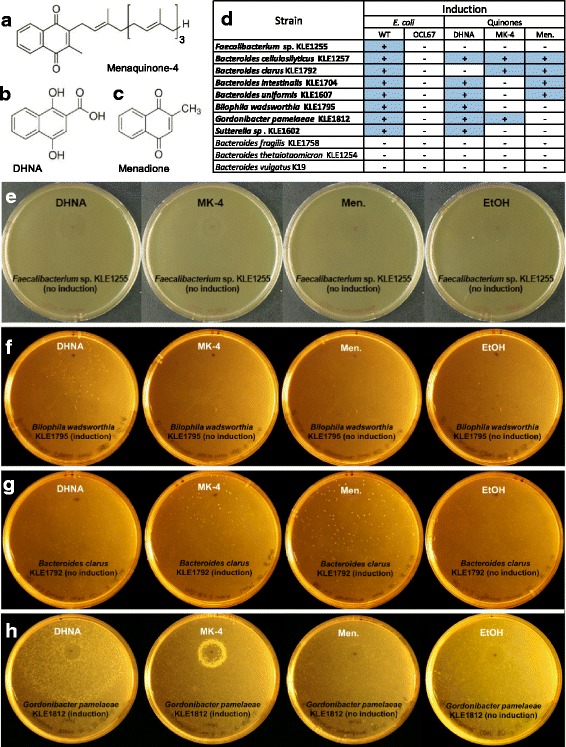
Quinones are growth factors for *E*. *coli-*induced bacteria. Bacteria were plated on rich BHIych agar and spotted with 5 μL of 1 mM of **a** MK-4, **b** DHNA, or **c** menadione, or the vehicle of the quinones, ethanol. The exception was KLE1255, which was spotted with 10 μL of 10 mM stocks all quinones. **d** *E*. *coli* and quinone induction capabilities of dependent strains and several cultivable *Bacteroides* species. Bolded strains represent induced organisms. Examples of induction with purified quinones here include **e** *Faecalibacterium* sp. KLE1255, **f** *Bilophila wadsworthia* KLE1795, **g** *Bacteroides clarus* KLE1792, and **h** *Gordonibacter pamelaeae* KLE1812

We similarly tested the other induced isolates identified in the co-culture screen and found that *E*. *coli* OCL67, deficient in menaquinone biosynthesis, was unable to stimulate their growth (Fig. 4d). As multiple *Bacteroides* species were found to be induced by *E*. *coli* in our original screening efforts, a panel of *Bacteroides* from our in-house strain collection was also tested for induction by OCL67, to explore whether this was a genus-wide phenomenon. Interestingly, these additional strains were not induced by *E*. *coli* but grew perfectly well on their own*,* suggesting induction can be species-specific (Fig. 4d).

### Quinones are growth factors for members of the gut microbiota

To test whether quinones were the growth factor for *Faecalibacterium* sp. KLE1255 and other isolates induced by *E*. *coli*, we evaluated three commercially available compounds, menaquinone-4 (MK-4), 1,4-dihydroxy-2-naphthoic acid (DHNA, a biosynthetic precursor of menaquinone), and menadione (a synthetic naphthoquinone analog that can act as a menaquinone precursor) (Fig. 4a–c). Suspensions of *Faecalibacterium* sp. KLE1255 and other organisms induced by *E*. *coli* were spread on solid medium, and each quinone was spotted on a separate plate (Fig. 4d–h). Addition of quinones enabled growth/induction of seven of the eight isolates tested (Fig. 4d). Interestingly, there was obvious specificity in the ability of quinones to support growth. For example, *Blautia wadsworthia* KLE1795 was induced only by DHNA (Fig. 4b); *Bacteroides clarus* KLE1792 was induced by MK-4 and menadione, but only weakly by DHNA (Fig. 4d, g); and *Gordonibacter pamelaeae* KLE1812 was induced by DHNA and MK-4, but not by menadione (Fig. 4d, h). The quinones did not induce growth of the *Bacteroides* strains we previously found not induced by *E*. *coli* (Fig. 4d).

Surprisingly, none of the three commercially available quinones induced the growth of *Faecalibacterium* sp. KLE1255 (Fig. 4d, e). Considering that this might be due to quinone specificity, as observed with the other strains, we tested additional quinones. However, no induction was observed when KLE1255 was grown in the presence of MK-5, MK-6, MK-7, MK-8, or ubiquinones—Q1, Q2, Q4, Q7, Q8, Q9, and Q10. Menaquinone and ubiquinone are very hydrophobic compounds, and it is possible that *E*. *coli* is helping with the delivery of these typically insoluble compounds. One possibility is that *E*. *coli* delivers its quinones encapsulated in outer membrane vesicles. An attempt to isolate the growth factor of *Faecalibacterium* sp. KLE1255 from *E*. *coli* was not successful, supporting the idea that *E*. *coli* secretes the quinone in a way to overcome solubility issues.

### Genome sequencing of *Faecalibacterium* sp. KLE1255 reveals a lack of a quinone biosynthetic pathway

**Fig. 5:**
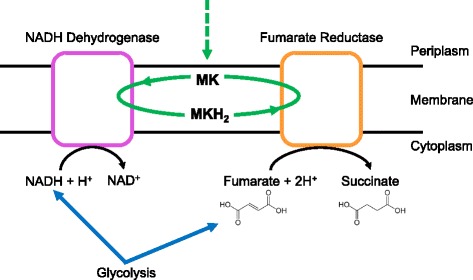
Model for menaquinone-dependence in *Faecalibacterium* sp. Genome analysis suggests that *Faecalibacterium* sp. possess a truncated electron transport chain that is directed towards fumarate respiration. The required menaquinone is provided by an external source (indicated by the dashed arrow). MK menaquinone, MKH_2_ menaquinol

Menaquinone is used in place of ubiquinone in the electron transport chain of some bacteria to transfer electrons during aerobic or anaerobic respiration. To determine the underlying mechanism of menaquinone-dependence of *Faecalibacterium* sp. KLE1255, we sequenced its genome and analyzed it for the presence of various pathways related to electron transport and respiration. KLE1255 does not have gene coding for menaquinone biosynthesis, and this is consistent with reports that no menaquinones could be detected from *Faecalibacterium* (formerly *Fusobacterium*) [22, 23]. We did find evidence for NADH dehydrogenase and fumarate reductase in the genome, suggesting that *Faecalibacterium* sp. KLE1255 possesses a truncated electron transport chain. No other anaerobic reductases were identified in the genome. When comparing the genome of *Faecalibacterium* sp. KLE1255 to other available *Faecalibacterium* sp. genomes [24], it became apparent that the components of the respiratory chain identified in these genomes were common among all strains. This may indicate that *F*. *prausnitzii* has a shortened electron transport chain that may be directed towards fumarate respiration while relying on externally provided menaquinone (Fig. 5).

## Discussion

In this study, we find that diverse members of the human microbiome can only grow (or have substantial growth improvement) in the presence of other gut bacteria. These bacteria come from different genera—*Bacteroides*, *Faecalibacterium*, *Bilophila*, *Gordonibacter*, *and Sutterella*. Different microorganisms, such as *Eggerthella lenta*, *Eubacterium rectale*, and several *Bacteroides* species, serve as “helpers” in these co-culture experiments, but surprisingly, we found that *E*. *coli* was able to support the growth of all induced bacteria we isolated. This enabled us to screen a library of *E*. *coli* gene knockout mutants for the genetic locus responsible for production of the growth factor. Notably, while these kinds of knockout libraries are not readily available for other intestinal bacteria, one could imagine creating and similarly screening an ordered transposon library to identify specific pathways producing growth factors in other helper species.

Unexpectedly, the screen pointed to a requirement for menaquinone biosynthesis in *E*. *coli* for it to serve as a universal helper. Menaquinone is a highly hydrophobic component of the anaerobic respiratory chain and would not be expected to serve as an exported growth factor. However, spotting purified menaquinone on a Petri dish produced a ring of growth of several, but not all *E*. *coli*-induced isolates. At the same time, a soluble precursor of menaquinone, DHNA, was a better inducer of growth, and superior to menadione, a synthetic precursor of quinones that is present in some growth media and was reported to be an essential growth factor for *Prevotella melaninogenicus* [25]. *P*. *melaninogenicus* does possess the genes *menA* and *ubiE*, which are required to convert DHNA into menaquinone. This suggests that DHNA, secreted by neighboring bacteria, may be the natural growth factor for *P*. *melaninogenicus* [25].

Our analysis shows that there is considerable specificity among uncultured bacteria for given quinone compounds, explaining why menadione is not a universal growth factor for these microorganisms. The observed specificity may be driven by variability in the capability to transport menaquinone (or the soluble quinone precursor DHNA); however, transporters for these compounds have not been described to date. Another possible explanation for the specificity profiles could be the capability to convert the menaquinone precursors and synthetic forms into their desired final quinones of choice. In this regard, *Faecalibacterium* sp. KLE1255 was the only isolate whose growth was not induced by adding purified quinones. Genome sequence of *Faecalibacterium* sp. KLE1255 showed a complete lack of quinone biosynthesis, but a capability to respire anaerobically. Given *Faecalibacterium* sp. KLE1255 does not possess the genes required to convert the precursors of menaquinone to a useable form (*menA* and *ubiE*), it likely requires a fully synthesized quinone not tested in our panel.

It is worth mentioning that the original isolation media for *Faecalibacterium prausnitzii* (formerly *Fusobacterium prausnitzii*) contained rumen fluid, which likely contains a variety of menaquinones produced by the ruminal microbiota [26, 27]. Furthermore, it has been reported that *F*. *prausnitzii* is unable to successfully monocolonize germ-free rats, but could when co-colonized with *Bacteroides thetaiotaomicron* [28] This, coupled with our findings*,* suggests that members of the *Faecalibacterium* genus are dependent on quinones from other bacteria to perform anaerobic respiration.

Why some anaerobic bacteria are stringently dependent on respiration is unclear. Indeed, the well-studied *E*. *coli* that can respire anaerobically is also capable of growing by fermentation. It is even more puzzling that a bacterium which requires respiration for growth would dispense production of an essential component of the electron transport chain, menaquinone. Our genomic analysis of *Faecalibacterium* sp. KLE1255 and the identification of other quinone-induced bacteria from the gut microbiota suggests widespread genetic loss of quinone biosynthesis. Recently, 254 genomes of gut bacteria were analyzed for co-occurrence of functional quinone biosynthesis pathways with anaerobic respiratory reductases [24]. One hundred out of 254 of the analyzed genomes had both a complete quinone biosynthetic pathway and terminal respiratory reductases, suggesting widespread respiration potential among the gut microbiota. A large number, nearly 25%, showed a pattern similar to what we found for *Faecalibacterium* sp. KLE1255—missing or incomplete menaquinone biosynthesis pathways, but the presence of at least one anaerobic respiratory reductase. The species that lost quinone biosynthesis appeared unrelated and included members of the genera *Akkermansia*, *Bacteroides*, *Bilophila*, *Clostridium*, and *Lactobacillus*, among others [24].

Notably, closely related type strains of several bacteria we identified in this study had a genetic signature of quinone dependence—an incomplete quinone biosynthesis pathway (Additional file 5: Table S3), yet genes encoding for predicted anaerobic respiratory reductases (Additional file 6: Table S4) [24]. At the same time, other species of the same genera as our quinone-induced isolates did retain the ability to produce their own quinones. For example, the type strain *Bacteroides vulgatus* ATCC 8482 has both a complete menaquinone biosynthesis pathway and anaerobic reductases (which is supported by the inability of the tested quinones to enhance growth of *B*. *vulgatus* K19). In the case of *Gordonibacter pamelaeae* KLE1812, which we originally isolated in co-culture with *Eggerthella lenta* and showed growth induction by quinones, there are > 20 anaerobic reductases based on the genome sequence of the type strain (*G*. *pamelaeae* DSM 19378). This underscores the need for quinones in bacteria that rely heavily on anaerobic respiration.

Our data suggest that the loss of quinone biosynthesis is an evolutionary recent event and happened independently in different lineages. Apparently, the presence of quinones in the gut allows for the loss of synthesis pathways and the appearance of such “cheaters.” The levels of menaquinones produced by *E*. *coli* or *B*. *vulgatus* in monocolonized rats are substantial, 6–8 μg/g [29], suggesting that these compounds are available to other bacteria. The loss of quinones by gut bacteria is analogous to the loss of siderophores we previously reported for marine uncultured bacteria which obtain them from neighboring species. In both cases, switching from expensive production of an essential component to acquisition will provide growth advantage. This scenario is consistent with the “Black Queen Hypothesis” [30], according to which loss of function at an individual level is favored if it is present among other members of the community.

## Conclusions

Our data suggest that menaquinones are a major class of required or growth-promoting factors for diverse bacteria from the human gut microbiome. This indicates loss of quinone biosynthesis happened independently in many lineages of the human microbiota. In addition to enabling access to novel diversity from the human microbiome via optimization of culture mediums, there is potential that supplementation of the microbiome with specific quinones or their efficient producers may enrich for desirable symbionts, such as *Faecalibacterium* species.

## Methods

### Human stool collection

Stool samples from an adult healthy human donor were collected using a commercially available stool collection vessel and with Northeastern University IRB approval. Within 5 min of collection, 1 g of stool was resuspended in 9 mL of sterile 20% glycerol in phosphate-buffered saline (PBS) and homogenized for 30 s using a vortex. One milliliter aliquots of this mixture was loaded in cryotubes and stored at − 80 °C. For cultivation experiments, individual aliquots of the frozen stool samples were then removed from the freezer and immediately transferred to a Coy Anaerobic Vinyl chamber with an atmosphere of 5% hydrogen, 10% CO_2_, and 85% nitrogen.

### Isolation and cultivation of helper-dependent bacteria

Serial dilutions of feces samples were spread-plated onto various rich medium (listed below). Plates were then incubated at 37 °C. All experiments were performed under strictly anaerobic conditions. Two different methods were used to obtain helper-dependent organisms: method A. The time of formation for all colonies were tracked for a week. Late-forming colonies (3–7 days) were diluted and spread-plated on fresh medium and nearby (< 5 mm), early-forming colonies (appearance after 1–2 days) were then resuspended in PBS at a high density and spotted (5 μL) on the sample plate. The co-cultures were incubated for up to 1 week in the chamber and observed daily. Growth induction of the dependent organism around the spotted helper indicated a positive hit. Method B: After spread-plating the feces samples, a helper spot of *Escherichia coli* K12 BW25113 was spotted on the same plate (5 μL). After incubation, colonies were picked in close proximity to the *E*. *coli* spot and tested for dependency on *E*. *coli* in the same way described for method A.

### Growth media

The following growth media were used in this study: brain heart infusion (BHI) supplemented with 5 g/L yeast extract, 1 g/L cysteine, and 15 mg/L hemin (only in agar) (BHIych); LYHBHI: BHI supplemented with 5 g/L yeast extract, 0.5 g/L cysteine, 5 mg/L hemin, 1 g/L cellobiose, 1 g/L maltose [16]; fastidious anaerobe agar (FAA); FAA with 5% (*v*/*v*) defibrinated sheep blood added (FAA blood); YCFAG (per liter): 4.5 g glucose (anhydrous), 10 g casitone, 2.5 g yeast extract, 4 g NaCHO_3_, 1 g cysteine, 0.45 g K_2_HPO_4_, 0.45 g KH_2_PO_4_, 0.9 g NaCl, 0.09 g MgSO_4_x7H_2_O, 0.09 g CaCl_2_, 1 mg resazurin, 10 mg hemin, 10 μg biotin, 10 μg hydroxycobalamin, 30 μg para-aminobenzoic acid, 50 μg folic acid, 150 μg pyridoxamine, 33 mM acetate, 9 mM propionate, 1 mM isobutyrate, 1 mM isovalerate, 1 mM valerate, 50 μg thiamine, 50 μg riboflavin, 15 g agar (optional)) [31]. Heat-labile vitamins were added after the medium was autoclaved. All media were prereduced overnight in an anaerobic chamber.

### Taxonomic assignment by 16S rRNA gene sequencing

PCR was performed using the general bacterial primers 27F (5’-AGAGTTTGATCMTGGCTCAG-3′) and 1492R (5’-TACGGYTACCTTGTTACGACTT-3′) to amplify part of the 16S rRNA gene. The PCR reaction mixture was 12.5 μL GoTaq Master Mix (Promega), 1 μL 10 μM 27F and 1492R primers (Operon), 9.5 μL Nuclease Free Water (Promega), and 1 μL of a colony resuspended in 100 μL sterilized distilled water. PCR was performed using an Eppendorf Mastercycler Personal, and the amplification conditions were one cycle of 95 °C for 5 min; 30 cycles of 95 °C for 30 s, 55 °C for 30 s, 72 °C for 90 s; and finally one cycle of 72 °C for 7 min. Amplification of PCR reactions were confirmed using gel electrophoresis on a 0.8% agarose gel containing ethidium bromide. Successful PCRs were sequenced by Macrogen Corporation using the 27F primer using the Applied Biosystems 3730xl DNA analyzer. Quality control for sequences was performed using DNA Baser (www.DnaBaser.com), in which ends were trimmed until there were more than 75% good bases (defined by having a QV score of higher than 25) in an 18-base window. Identification of phylogenetic neighbors and calculations of pairwise sequence similarity were done using the EZTaxon server (http://www.eztaxon.org). 98.7% similarity by 16S was used to determine species level cutoff using a previously suggested level [32].

### Screening *E*. *coli* deletion mutants for a lack of growth induction

For the library screen, a selection of 283 strains from the *E*. *coli* small-, medium-, and large-scale deletion libraries were screened [20, 21]. The mutants were obtained from the Japanese National BioResource Project. The *E*. *coli* strains were grown anaerobically overnight in BHIych. The next day, KLE1255 was plated onto BHIych agar and 1 μL spots of *E*. *coli* cultures were added to the plates. After 2 days of incubation, plates were evaluated for growth of KLE1255. All other induction assays were performed using *E*. *coli* suspensions, prepared in phosphate-buffered saline or growth medium, rather than overnight cultures as no difference was observed for the different treatments.

### Quinone induction experiments

Inoculums of strains were either created by picking colonies from agar plates seeded with *E*. *coli* K12 BW25113 or from 24 to 48 h cultures in BHIych. These inocula were then bead spread on solid BHIych until dried, and 5 μL of a 1-mM stock of DHNA, MK-4, or menadione (all purchased from Sigma and resuspended in 100% ethanol) was spotted, with ethanol as a control. For *Faecalibacterium* sp. KLE1255, concentrations of up to 10 mM of DHNA, MK-4, or menadione were also tested. An expanded panel of quinones were also tested for *Faecalibacterium* sp. KLE1255 induction: MK-8 purified from *E*. *coli* K12 BW25113, and MK-4, MK-5, MK-6, MK-7, and MK-8 purified from *Micrococcus luteus* KLE1011. Ubiquinones tested include Q1, Q2, Q4, Q9, and Q10 (all available from Sigma-Aldrich) as well as Q7 and Q8, which were purified from *E*. *coli*. These were tested using multiple solvents (acetone, MeOH, DMSO, and hexane) at concentrations of 10 mg/mL, 1 mg/mL, and 0.1 mg/mL and 10 μL spotted. Methods for isolation of the expanded quinone panel are described below.

### Isolation of quinones

For the expanded quinone panel, quinones were isolated from *Escherichia coli* K12 BW25113 and *Micrococcus luteus* KLE1011. Strains were streaked on plates of BHIych or R2Asea, respectively, and individual colonies were picked to inoculate 5.0 mL cultures, which were grown for 24 h at 250 rpm. These starter preps were used to inoculate 1.0 L cultures, which were grown for 24 h at 160 rpm. A batch of eight, 1.0 L cultures was routinely prepared for each organism for cell pellet and supernatant extractions. One liter cultures of bacteria were centrifuged in a Beckman Coulter Avanti J-20 XP centrifuge equipped with a JLA 8.1000 rotor at 4 °C. Cells were pelleted at 4500 rpm (5053×*g*) for 20 min. The supernatant was poured off and the cells were washed with 1% NaCl. The cells were pooled and pelleted at 6000 rpm (8983×*g*) for 20 min. The supernatant was discarded, and the cells were submitted to quinone extraction. Quinones were isolated either via extraction of saponified cells or via direct solvent extraction of pelleted cells. Saponification was performed in line with established protocols [33]. Briefly, pelleted cells (from 8 × 1.0 L preps) were suspended in a 150 mL, 3:2:1 mixture of ethanol:water:25% KOH containing 2.5 g pyrogallol, transferred to a round bottom flask and refluxed for 20 min at 100 °C under an inert atmosphere of dry gas (nitrogen or argon) and under the exclusion of light while stirring vigorously. The mixture was immediately cooled to room temperature in an ice-water bath and then extracted 4× with equivolume portions of heptane. The organic layers were pooled, dried over anhydrous Na_2_SO_4_, filtered, and concentrated to dryness in vacuo. The dried, oily material was stored under inert gas (nitrogen or argon) at − 20 °C until chromatographic purification was performed. Direct solvent extraction was performed by suspending pelleted cells (from 8 × 1.0 L preps) in 300 mL of a 3:1 ethanol:diethyl ether solution, transferring to a round bottom flask and stirring vigorously for 2.5 h under an atmosphere of dry, inert gas and under the exclusion of light. The suspension was filtered, the cells were rinsed with a portion of diethyl ether, and the filtrate was dried to 25% its original volume in vacuo. The concentrate was diluted with water and extracted 3× with 500-mL portions of heptane. The pooled organic layer was then dried and stored as described above. Quinones and menachromenols were purified using an Agilent Technologies 1200 Series High Performance Liquid Chromatography system equipped with G1361A Prep Pumps and a G1315D diode array detector. Samples were prepared in 2.0 mL of acetonitrile and were purified on a Phenomenex Luna C8(2), 250 × 21.20 mm, 5 mm reverse phase HPLC column. Material was eluted using an isocratic solvent system consisting solely of HPLC grade acetonitrile at a 10-mL min^−1^ volume flow rate.

### NMR characterization of isolated quinones

All NMR spectra of bacterially derived compounds were acquired using a CDCl_3_ susceptibility matched 5-mm Shigemi® NMR tube on a Varian VNMRS 600-MHz NMR spectrometer equipped with a 5-mm HCN AutoX inverse probe. Data was acquired using VnmrJ version X software and was analyzed using MestReNova version 7.0.0-8331 software. Trace acid was removed from “100%” CDCl_3_ solvent (99.96 atom % D–Aldrich) by passing over a plug of Brockmann grade I activated, basic aluminum oxide (Aldrich) immediately before use. Spectra were referenced to TMS or residual protio solvent.

### Isolation of genomic DNA from Faecalibacterium sp. KLE1255 for whole genome sequencing

A phenol/chloroform prep was performed to obtain genomic DNA. Three milliliter of a turbid culture were pelleted and resuspended in 500-μL lysis buffer (20 mM Tris 7.5; 50 mM EDTA; 100 mM NaCl). Fifty microliter of 20 mg/mL freshly prepared lysozyme and 100 μg/mL proteinase K were added and the mixture. After 30–60-min incubation, 60 μL 10% (*w*/*v*) sarkosyl (N-lauroylsarkosine) was added and vortexed. Next, 600 μL TE-saturated phenol was added; the contents vigorously vortexed for 15 s and centrifuged for 5 min at 13,000 rpm. Then, the aqueous phase was removed to a new tube and 600 μL phenol/chloroform were added, the mixture vortexed and the aqueous phase removed to a new tube again. One-tenth volume (60 μL) of 3 M NaOAc was added, mixed, and two volumes EtOH were added. The tube was inverted until the DNA precipitated (4–6 times). The DNA was centrifuged down for 1 min and the supernatant removed. One-hudred fifty microliter of 70% (*v*/*v*) EtOH were added and vortexed, and the DNA centrifuged again. The supernatant was removed and the pellet air-dryed for 10–15 min. Finally, the DNA was resuspended in 100 μL elution buffer (10 mM Tris-Cl, ph 8.5).

### Whole genome sequencing and annotation of *Faecalibacterium* sp. KLE1255

Five microliter of genomic DNA was sent to George Weinstock at The Genome Institute at Washington University, St. Louis, Missouri, for Illumina sequencing. The draft genome, consisting of 119 contigs, was annotated using the RAST (Rapid Annotation using Subsystem Technology) server [34] and the KEGG (Kyoto Encyclopedia of Genes and Genomes) database.

## Additional files

Additional file 1: Table S1. Entire chromosome *E. coli* deletion library reformatted. 283 strains were first compiled from *E. coli* small-, medium-, and large-scale deletion libraries to cover all non-essential genes of the *E. coli* genome. Strains were taken from the Keio collection [19] and two larger deletion libraries [20, 21]. (XLS 36 kb)

Additional file 2: Table S2. Strains identified in the *E. coli* knockout screen unable to induce the growth of KLE1255. Includes information on which genes are absent for each clone. (XLSX 18 kb)

Additional file 3: Figure S1. Single deletions in the *E. coli* chorismate biosynthesis pathway prevented growth induction of KLE1255*.* Single deletion mutants for all genes involved in chorismate biosynthesis were tested for induction capabilities of KLE1255. Red boxes indicate *E. coli* mutants with impaired growth induction capabilities for KLE1255. (PNG 449 kb)

Additional file 4: Figure S2. Single deletions in the *E. coli* menaquinone-8 pathway, but not ubiquinone-8 pathway, prevented growth induction of KLE1255*.* Single deletion mutants for all genes involved in ubiquinone-8 and menaquinone-8 biosynthesis were tested for induction capabilities of KLE1255. Red boxes indicate *E. coli* mutants with impaired growth induction capabilities for KLE1255. (PNG 364 kb)

Additional file 5: Table S3. Quinone-induced bacteria have a disrupted menaquinone biosynthesis pathway, while related organisms not induced by quinones have a complete pathway. The genomes of the nearest type strains of all *E. coli*- or quinone-induced cultured bacteria were surveyed manually for the presence of a functional menaquinone biosynthesis pathway using a published dataset [24]. All organisms induced by *E. coli* or quinones in earlier co-culture experiments were missing large components of the menaquinone biosynthesis pathway, while *Bacteroides* species not induced by *E. coli* or quinones were predicted to have complete menaquinone biosynthetic capabilities. No strains were found to have predicted copies of genes in the futalosine pathway, an alternative means to generate menaquinone. *ubiE/menG:* 2-methoxy-6-polyprenyl-1,4-benzoquinol methylase; *menF* = Menaquinone-specific isochorismate synthase; *menD* = 2-succinyl-5-enolpyruvyl-6-hydroxy-3-cyclohexene-1-carboxylic-acid synthase; *menH* = 2-succinyl-6-hydroxy-2,4-cyclohexadiene-1-carboxylate synthase; *menY* = 2-succinyl-5-enolpyruvyl-6-hydroxy-3-cyclohexene-1-carboxylate dehydrogenase; *menC* = o-succinylbenzoate synthase; *menE* = o-succinylbenzoic acid--CoA ligase; *menB* = Naphthoate synthase. *menI* = 1,4-dihydroxy-2-naphthoyl-CoA hydrolase; *menJ* = 1,4-dihydroxy-2-naphthoyl-CoA hydrolasein (putative); *menA* = 1,4-dihydroxy-2-naphthoate polyprenyltransferase; *mqnA* = Chorismate dehydratase; *mqnE* = Aminodeoxyfutalosine synthase; *mqnC* = Cyclic dehypoxanthine futalosine synthase; *mqnD* = 1,4-dihydroxy-6-naphthoate synthase; *mqnZ* = 1,4-dihydroxy-6-naphthoate synthase (alternative); *mqnX* = Aminodeoxyfutalosine deaminase; *mqnB* = Futalosine hydrolase (EC 3.2.2.26); *mtnN* = Aminodeoxyfutalosine nucleosidase; *mqnL* = 1,4-dihydroxy-6-naphthoate carboxy-lyase, UbiD-like; *mqnM* = 2-heptaprenyl-1,4-naphthoquinone methyltransferase; *mqnP* = 1,4-naphthoquinone polyprenyltransferase. Data was taken and modified from Racheev, 2016. (XLSX 10 kb)

Additional file 6: Table S4. Quinone-dependent and control strains have predicted anaerobic reductases. The genomes of nearest type strains of all *E. coli*- or quinone-induced cultured bacteria were surveyed manually for the presence of individual annotated anaerobic reductases using a published dataset [35]. All analyzed organisms have the genetic capability to utilize anaerobic reductases for anaerobic respiration. Arx = Arsenate reductase; Cyd = Cytochrome bd reductase; Dms = Dimethyl sulfoxide reductase; Dsr = Sulfite reductase; Frd = Fumarate reductase; Nap = Nitrate reductase; Nar = Nitrate reductase; Nrf = Nitrite reductase; Phs = Thiosulfate reductase; Psr = Polysulfite reductase; Tor = Trimethylamine N-oxide reductase; Ttr = Tetrathionate reductase; Ynf = Selenate reductase. Data was taken and modified from Racheev, 2014 [35] and Ravcheev, 2016 [24]. (XLSX 8 kb)

## Abbreviations

BHI: Brain heart infusion
BHIYCH: Brain heart infusion supplemented with 5 g/L yeast extract, 1 g/L cysteine and 15 mg/L hemin
CFU: Colony-forming units
DHNA: 1,4-Dihydroxy-2-naphthoic acid
FAA: Fastidious anaerobic agar
LYHBHI: BHI supplemented with 5 g/L yeast extract, 0.5 g/L cysteine, 5 mg/L hemin, 1 g/L cellobiose, 1 g/L maltose
MK#: Menaquinone; # = length of the isoprenoid sidechain
PBS: Phosphate-buffered saline
Q#: Ubiquinone; # = length of the isoprenoid sidechain
