# Supplementary figures and images for: Quinones are growth factors for the human gut microbiota
Source: Microbiome. 2017 Dec 20;5:161. doi: 10.1186/s40168-017-0380-5 (PMC5738691; doi:10.1186/s40168-017-0380-5)

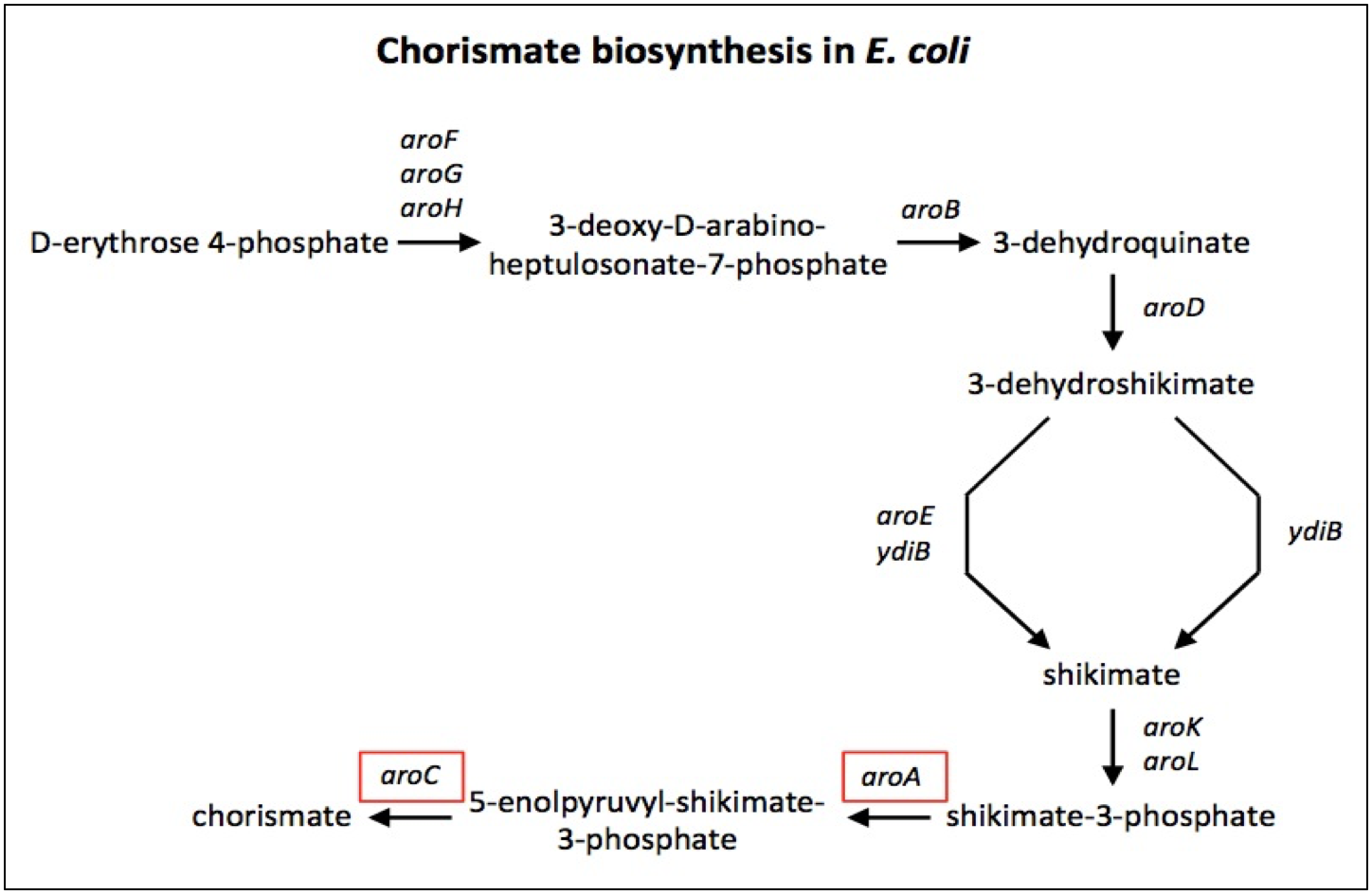

Supplement: Supplementary file 3 — Single deletions in the E. coli chorismate biosynthesis pathway prevented growth induction of KLE1255. Single deletion mutants for all genes involved in chorismate biosynthesis were tested for induction capabilities of KLE1255. Red boxes indicate E. coli mutants with impaired growth induction capabilities for KLE1255. (PNG 449 kb) [file 40168_2017_380_MOESM3_ESM.png]

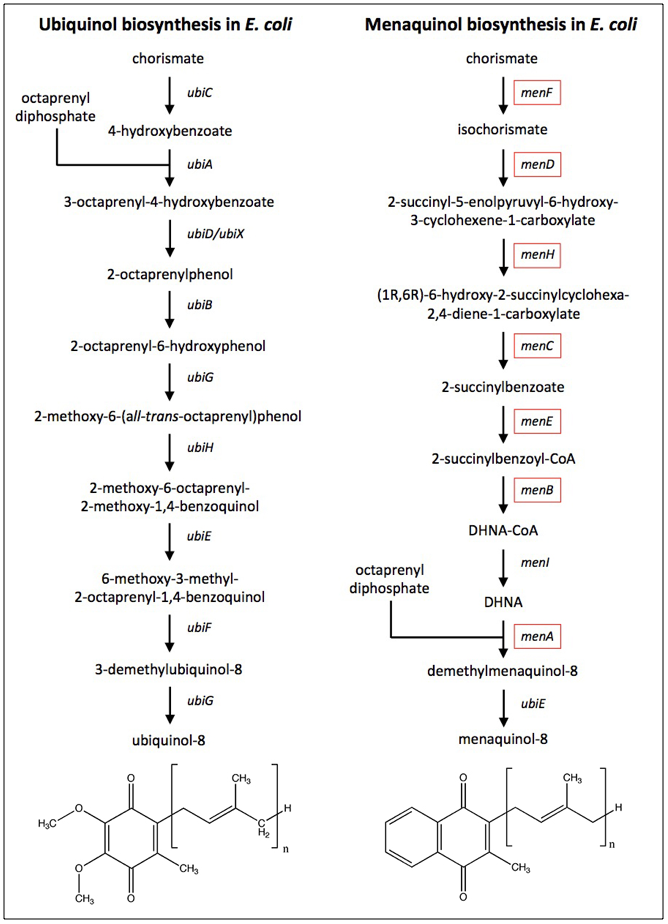

Supplement: Supplementary file 4 — Single deletions in the E. coli menaquinone-8 pathway, but not ubiquinone-8 pathway, prevented growth induction of KLE1255. Single deletion mutants for all genes involved in ubiquinone-8 and menaquinone-8 biosynthesis were tested for induction capabilities of KLE1255. Red boxes indicate E. coli mutants with impaired growth induction capabilities for KLE1255. (PNG 364 kb) [file 40168_2017_380_MOESM4_ESM.png]
